# Natural Products from Cyanobacteria: Focus on Beneficial Activities

**DOI:** 10.3390/md17060320

**Published:** 2019-05-30

**Authors:** Justine Demay, Cécile Bernard, Anita Reinhardt, Benjamin Marie

**Affiliations:** 1 UMR 7245 MCAM, Muséum National d’Histoire Naturelle-CNRS, Paris, 12 rue Buffon, CP 39, 75231 Paris CEDEX 05, France; justine.demay1@mnhn.fr (J.D.); benjamin.marie@mnhn.fr (B.M.); 2 Thermes de Balaruc-les-Bains, 1 rue du Mont Saint-Clair BP 45, 34540 Balaruc-Les-Bains, France; anita.reinhardt@thermesbalaruc.com

**Keywords:** cyanobacteria, natural products, metabolites, biological activities, producers, chemical classes

## Abstract

Cyanobacteria are photosynthetic microorganisms that colonize diverse environments worldwide, ranging from ocean to freshwaters, soils, and extreme environments. Their adaptation capacities and the diversity of natural products that they synthesize, support cyanobacterial success in colonization of their respective ecological niches. Although cyanobacteria are well-known for their toxin production and their relative deleterious consequences, they also produce a large variety of molecules that exhibit beneficial properties with high potential in various fields (e.g., a synthetic analog of dolastatin 10 is used against Hodgkin’s lymphoma). The present review focuses on the beneficial activities of cyanobacterial molecules described so far. Based on an analysis of 670 papers, it appears that more than 90 genera of cyanobacteria have been observed to produce compounds with potentially beneficial activities in which most of them belong to the orders Oscillatoriales, Nostocales, Chroococcales, and Synechococcales. The rest of the cyanobacterial orders (i.e., Pleurocapsales, Chroococcidiopsales, and Gloeobacterales) remain poorly explored in terms of their molecular diversity and relative bioactivity. The diverse cyanobacterial metabolites possessing beneficial bioactivities belong to 10 different chemical classes (alkaloids, depsipeptides, lipopeptides, macrolides/lactones, peptides, terpenes, polysaccharides, lipids, polyketides, and others) that exhibit 14 major kinds of bioactivity. However, no direct relationship between the chemical class and the respective bioactivity of these molecules has been demonstrated. We further selected and specifically described 47 molecule families according to their respective bioactivities and their potential uses in pharmacology, cosmetology, agriculture, or other specific fields of interest. With this up-to-date review, we attempt to present new perspectives for the rational discovery of novel cyanobacterial metabolites with beneficial bioactivity.

## 1. Introduction

Cyanobacteria belong to an ancient group of photosynthetic prokaryotes that present a very wide range of cellular strategies, physiological capacities, and adaptations that support their colonization of very diverse microenvironments worldwide. As a consequence, cyanobacteria occur in varied and often extreme habitats and are then able to settle in diverse biotopes (e.g., marine, terrestrial, freshwater, thermal springs) [1,2,3]. They are also well known for the production of a wide variety of bioactive natural products, including some potent toxins (e.g., microcystins, anatoxins, and saxitoxins) [2,3]. Due to the remarkable capability of cyanobacteria to proliferate and form toxic blooms that induce potential human health consequences [4], numerous studies have been conducted to develop tools for the monitoring of such blooms [5,6] or effective strategies for the mitigation of their overgrowth [7]. On the contrary, certain cyanotoxins could also constitute a promising opportunity for drug development such as certain cancer therapies [8].

Two main aspects known as the chemical diversity and the related bioactivity have to be considered when considering the application potential of natural products produced by cyanobacteria. The chemical diversity of metabolites produced by these organisms has been well described and about 15 reviews have been already published in the past 20 years, dealing with their structural and chemical diversity [9,10,11,12,13,14] or their respective biosynthetic pathways [15,16]. Beyond the notorious harmful effects of cyanotoxins, other cyanobacterial natural products show a wide range of bioactivities that could be potentially useful for diverse applications [17,18,19,20,21]. So far, among the existing reviews related to the diversity of cyanobacterial metabolites, only one has addressed the relative taxonomical positions of the different producing strains [9]. A few taxa appear to be especially prolific producers of a large set of metabolites, while others still remain to be investigated. Recent genomics approaches and genome sequencing have been important steps in the elucidation of the pathways implicated in the biosynthesis of natural products. Their wide structural diversity has been described as a consequence of the numerous biosynthetic pathways developed by cyanobacteria in order to produce these metabolites [15]. Most of the active cyanobacterial molecules are considered as being produced either through the non-ribosomal peptide (NRP) or the hybrid polyketide-NRP biosynthetic pathways [10], or by the ribosomal synthesis of pro-peptides that are post-translationally modified (RiPP). Previous genome analysis demonstrated that the diversity of the known metabolites is merely a fraction of the true metabolic potential of cyanobacteria [15]. Concerning bioactivity, cyanobacteria have long been a source of molecules with a potent nutritional property [18]. Aztec civilizations consumed cyanobacteria (*Spirulina*) in their routine diet [22], and Chadian populations still use them as one of their substantial food sources [23]. Besides nutritional and probiotic purposes [13,21], cyanobacteria are well-known as an important source of metabolites with technological applications in the biotechnical or pharmaceutical fields, which lead to an increase in interest in these research realms [10]. Most bioactivities described to date are the antibacterial, antifungal, anti-cancerous, immunosuppressive, anti-inflammatory, and anti-tuberculosis activities that have the potential to be used in fields such as pharmacology, cosmetology, agriculture, the food industry, or as biofuel [17]. Cyanobacteria cells represent a sustainable resource for biotechnology due to their photosynthetic, N-fixation, and autotrophic capacities [17,18,24]. Due to the current increase in their pharmaceutical value and in their application prospects for use in medicine or biotechnology, the exploration of uncovered cyanobacterial taxa constitutes a promising strategy to efficiently explore the chemical diversity of their bioactive compounds.

The present review globally and systematically describes current knowledge on the biological activities described for cyanobacterial natural products, and, thanks to the construction of a specific and freely available molecular database, regroups all information described so far concerning the chemical structures, the producing organisms, and the various bioactivities of all the different cyanobacterial metabolite families. This original material allows us to depict, from data based on exhaustive literature, which kinds of bioactive metabolite are potentially produced by the different cyanobacterial taxa. In this case, the producer organisms were considered at different taxonomic levels (family, order, and genus) and are referenced according to their original habitats (freshwater, marine, and others). The chemical diversity is described with respect to the different kinds of bioactivity and the potential links between them are questioned, according to their potential or effective molecular mechanisms of action. A specific focus on 47 cyanobacterial compounds presenting beneficial bioactivities is detailed and discussed regarding their potential in pharmaceutical, cosmetical, biotechnical, and agricultural applications, which opens new perspectives on the discovery of novel and potent bioactive cyanobacterial molecules.

## 2. Methods for Dataset Construction

A database was constructed using different search engines, notably PubMed and Google Scholar. The keywords used were “cyanobacteria,” “metabolite” or “natural product,” “beneficial” and “activity,” or “biological properties.” The database was first based on reviews and further completed with recent publications dealing with the isolation of new compounds from cyanobacteria. 

The main entries into the database were the names of the metabolites. To avoid bias in the counting of metabolites, we stored all the data for each molecule and its variants as a “family.” In fact, there are still no molecular classification references for a natural product description. As discussed by Janssen [25], there is no standardized naming system along cyanobacterial metabolites, as in natural product discovery in general, that could induce an underestimation of the real diversity of natural products and to hide the potential link between their chemical structures, biosynthetic pathways, and evolution routes. Thus, such a valuable classification of cyanobacterial metabolites is still needed, notably in the current context of genomic and metabolomic development.

In our database, metabolites were grouped and classified based on different criterion, initially selected by different authors [13,15,25]. First, they were classified according to their biosynthetic pathways based on the genomic data reviewed by Dittmann et al. (e.g., microcystins, cryptophycins, and aeruginosins) [15]. Secondly, when biosynthetic information was not available, metabolites were classified, according to their structural homology, as proposed by Boudreau et al., [26], Janssen [25], and Chlipala et al. [13], supposing that they might be sharing at least a similar, if not the same, biosynthetic route (e.g., kulolide-family, aerucylamides, and cyanopeptolins). In most cases, metabolite variants have a few differences occurring on a few residues and have conserved the specific structure of their metabolite family. For example, the cyanopeptolin-like family, which contains, so far described, 139 variants, is comprised of a core structure of six amino acid residues and a variable side chain containing between 1 and 3 residues. The sequence of the amino acids in the core structure is usually composed with: Thr − [Leu|Arg|Tyr] − Ahp − [Ile|Phe|Thr|Leu|Val] − N-Me[Tyr|Phe] − [Val|Ile] (see Supplementary Data S1). Some amino acids are variable (in brackets) and some others are identical in the large majority of the variants, notably the 3-amino-6-hydroxy-2-piperidone (Ahp), and the threonine (Thr) that support the side chain and close the cycle with an ester bond linkage (S1) [13].

The data collected were then classified depending on the chemical class of the compound, the chemical structure, and the strain producing the metabolites with all the taxonomic information (species, genus, family, and order), in accordance with Komarek et al. (2014) [27]. In addition, we compiled the demonstrated activities for the purified compounds. Fourteen classes of activity were mostly tested through the literature: lethality (against brine shrimp, and other small invertebrates), neurotoxicity, hepatotoxicity, dermal toxicity, cytotoxicity, anti-inflammatory activity, antioxidant activity, antiviral, antibacterial, antifungal, antialgal, antiprotozoal, serine protease inhibition, and other types of enzyme inhibition.

**Figure 1:**
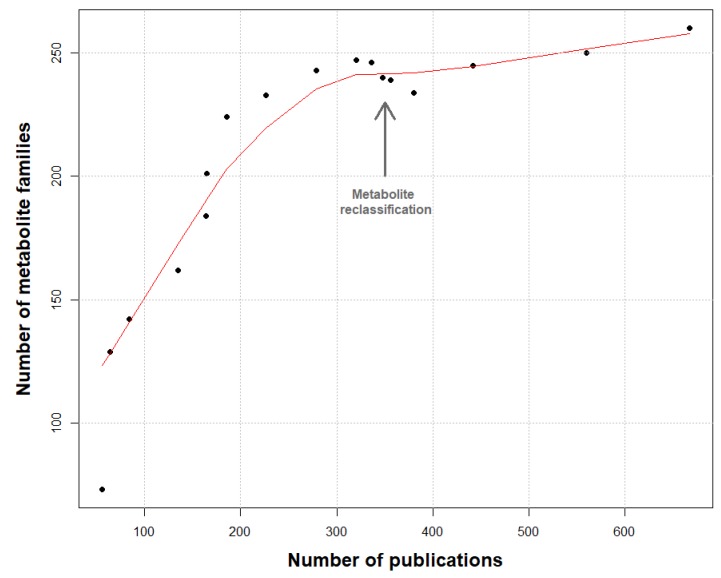
Evolution of the cumulative number of metabolite families according to the number of analyzed publications used for the construction of the database. The arrow indicates a reclassification event of all the structural variants of one molecule in a unique entry of “family” [13,25,26]. We observed a progressive stabilization of the number of compound families in the database that supports the postulation of the exhaustiveness of the present database.

Additionally, 670 publications were analyzed, dating from the 1970s until today (April 2019). Around 1630 unique molecules have been reported so far and were grouped in 260 families of metabolites (see Supplementary Data S2). To validate the knowledge depth of our work, a rarefaction curve of the number of molecule families was constructed using the number of analyzed publications (Figure 1).

## 3. Taxonomy of the Producing Strains

**Figure 2:**
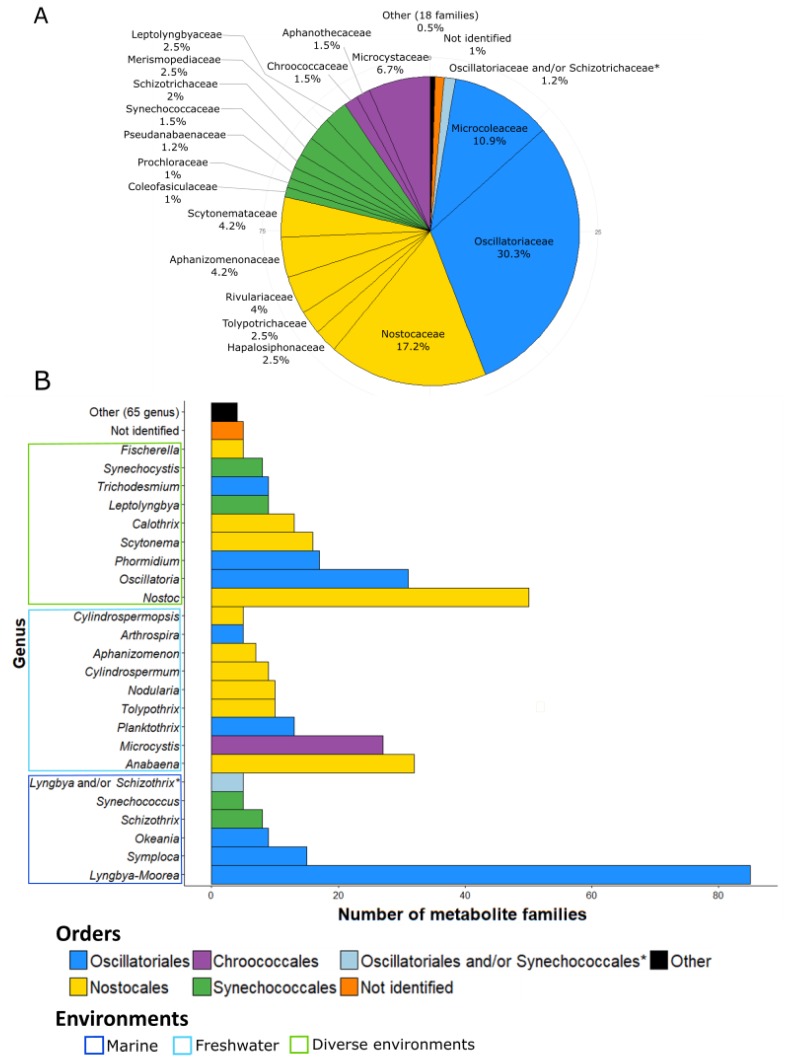
Proportion of families of compound by taxonomical level. (**A**) The pie chart represents the percentage of compound families for each taxonomical family. Note that some compound families can be produced by several cyanobacterial families. The “Other” category concerns other taxonomical families that produce less than two compound families. (**B**) The histogram shows the number of compound families for each genus. The “Other” category corresponds to genera producing less than four compound families. * indicates cyanobacterial assemblages whom the real metabolite producer is still undetermined. The boxes indicate the environmental origins for the corresponding genera. For both charts, the colors correspond to the taxonomical order of each genus or family.

The 260 families of molecules were attributed to cyanobacteria at their different taxonomic levels (order, family, and genus) (Figure 2). Some families of compounds can be produced by different strains and, thus, occur at different taxonomical levels. For example, microcystins are produced by various strains belonging to seven different genera, five families, and three orders.

The Oscillatoriales produces the largest number of metabolite families (153 families, 46.5%). The strains belonging to the Nostocales are also considerable producers of metabolites with 98 families (29.7%). The other main producers are the strains belonging to Chroococcales and Synechococcales, which exhibit, respectively, 34 and 31 described molecule families (10.3% and 9.4%). It is interesting that, except for these four orders, the others (i.e., Pleurocapsales, Chroococcidiopsales, Gloeobacterales, and Spirulinales) remain weakly represented in the database: less than five families of metabolite have been reported so far for all of them.

Some metabolites have been isolated from cyanobacterial assemblages without accurate identification of the producer organisms. For these cases, the authors identified the genera of the two dominant cyanobacteria of the assemblage but could not accurately determine which one of them produces which molecule [28,29,30,31,32,33,34,35,36,37,38,39,40]. Tidgewell et al. (2010) [9] also identified the prevalence of marine cyanobacterial products within Oscillatoriales and Nostocales with 58% and 24% of the isolated molecules, respectively. Within Oscillatoriales, members of the genus *Lyngbya*, and, notably, *Lyngbya majuscula* produce the highest number of metabolites. This benthic genus is widely spread through the tropical marine ecosystem and has been widely studied because of its toxicity and implication in many dermatitis cases around the world [41,42]. A number of studies have been conducted on the *Lyngbya* genus, and a high number of new metabolites have been described. In fact, *Lyngbya* is, to date, the most productive genus of bioactive cyanobacterial compounds (Figure 2B). Recent studies showed that *Lyngbya* is polyphyletic [27,43] and using polyphasic approaches, *Lyngbya* has been split into four new genera: *Moorea* [44], *Okeania* [45], *Limnoraphis* [46], and *Microseira* [47]. Some marine strains previously identified morphologically as *Lyngbya majuscula* and *Lyngbya sordida* were, therefore, renamed as *Moorea producens*, and some strains of *Lyngbya bouillonii* were renamed to *Moorea bouillonii* on the basis of molecular and phylogenetic analyses [44]. In the same way, some freshwater strains morphologically identified as *Lyngbya wollei* were separate from the *Lyngbya* genus and described as *Microseira wollei* after analysis of their phylogenetic position [47].

According to this information, we decided to present the number of metabolite families produced by the *Lyngbya* and the *Moorea* genera together (reported as *Lyngbya-Moorea* in Figure 2B), given that the majority of families isolated from *Lyngbya* species were reported to be from *Lyngbya majuscula* (46 of 78 described from all the *Lyngbya*) or from *Lyngbya* spp. strains sampled from tropical marine environments (22 of 78), as described for the *Moorea* genus and were possibly misidentified with regard to this newly described genus [44].

At the family level, the main producers of known bioactive compounds belong to Oscillatoriaceae (30.3%, producing 122 families of compounds), followed by Nostocaceae and Microcoleaceae (17.2% and 10.9% for 69 and 48 molecule families, respectively) (Figure 2A). At the genus level (Figure 2B), *Lyngbya-Moorea* exhibits the highest number of isolated compounds (85 families of metabolites representing 20.6%), in accordance with the perceived richness of production for the *Lyngbya* genus due to its polyphyletic status [48]. *Nostoc* is the second most prolific genus of bioactive compound families with 50 isolated families so far (12.1% of the total number of families of metabolites). The other most important genera are *Anabaena*, *Oscillatoria*, and *Microcystis* (with 32, 31, and 27 families of molecules, respectively, representing 7.8%, 7.5%, and 6.6%) (Figure 2B).

When looking at the habitats of these cyanobacteria, a large number of compounds were isolated from marine environments (148 families of metabolite in the database, which means 53% of the families of metabolites) in comparison to the number of strains isolated from freshwater environments (77 families of metabolites, 27.6%) (Figure 2B). However, this difference might be at least partly due to the high number of compounds isolated from the marine species *Lyngbya majuscula-Moorea producens* (49 families of molecules, 18.8% of the families in the database) and to the existence of various research programs focused on marine species (e.g., the Panama International Cooperative Biodiversity Group, ICBG). 

Overall, we observed that diversity at the genus level is important, as illustrated by the 90 different genera present in the database. Moreover, 65 different genera have been reported to produce less than four molecules (Figure 2B). We also noticed that five molecules were isolated from *Lyngbya*/*Schizothrix* assemblages and five others from unidentified strains of cyanobacteria (Figure 2B). Thus, at the genus level, the diversity of producers is large with a high number of genera studied (90 different genera). Nevertheless, these genera generally belong to the same orders (e.g., Oscillatoriales, Nostocales, Synechococcales, and Chroococcales) while some orders were not studied. For example, among the Pleurocapsales order, only four genera have been reported to produce metabolites. As a result, the covered diversity appears not to be exhaustive and can still be increased.

According to Shih et al. (2013) [49], the genomic potential of cyanobacteria to produce secondary metabolites is high with more than 70% of the studied strains presenting non-ribosomal peptide synthase (NRPS) or polyketide synthase (PKS) gene clusters in their genomes. In particular, they identified one strain belonging to the *Fischerella* genus (*Fischerella* sp. PCC 9339) that exhibits 22 NRPS/PKS clusters in its genome. On the contrary, only five compound families have been isolated from the genus *Fischerella* so far and are listed on the present database. Moreover, it is interesting to note that, among the 126 strains analyzed by Shih et al. (2013) [49], only 14 were formally reported to produce characterized metabolites.

In addition, the best producer genus, *Lyngbya-Moorea*, remains rarely studied at the genomic level: four genomes are available in the Genbank database and another three are available on the Microscope platform [50]. Considering the number of compounds isolated from the *Lyngbya-Moorea* genus (85 compound families), most of the links between the identified molecules and the responsible biosynthetic gene clusters remain to be characterized. We also compared our collected data with those reported by Dittman et al. (2015) [15] in order to determine when the isolated molecule families are linked with a specific and identified biosynthetic gene cluster. This review showed that less than 20% of the molecule families from the database are associated with specific identified biosynthetic gene clusters. Thus, the biosynthetic pathways of a large majority of compounds is still unknown as well as the regulation mechanisms controlling their production. Therefore, these observations highlight part of the remaining possibilities for the discovery of new molecules, gene production, and biosynthetic pathways.

## 4. Chemical Diversity and Bioactivity of Natural Products from Cyanobacteria

**Figure 3:**
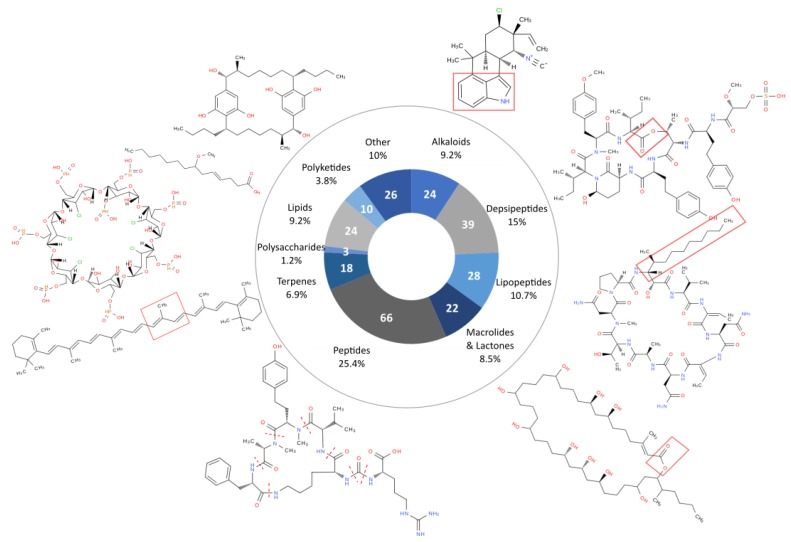
Classification of the 260 cyanobacterial metabolite families according to their respective chemical classes. All the molecules have been classified into these different classes according to their respective structural characteristics. For example, the depsipeptides are a class of peptides containing an ester bond and macrolides are molecules exhibiting a macrocycle and one or more lactone functions. Some examples of cyanobacterial molecules belonging to these classes are illustrated. Hapalindole A (alkaloids), Oscillapeptin A (depsipeptides), Minutissamide A (lipopeptides), Caylobolide B (macrolides/lactones), Anabaenopeptin E (peptides), β-carotene (terpenes), Cyclodextrin phosphate (polysaccharides), Lyngbic acid (lipids), and Cylindrocyclophane A (polyketides). The main characteristics of each chemical class are highlighted in red. All the structures were obtained from the ChEMBL Database (https://www.ebi.ac.uk/chembl/).

**Figure 4:**
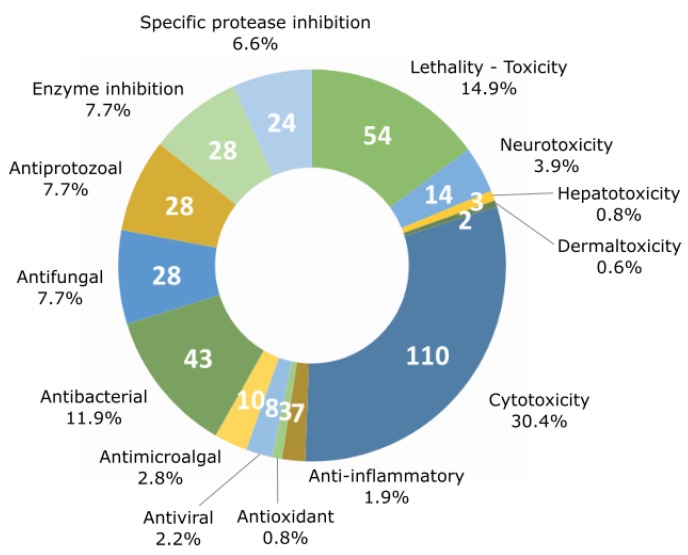
Number of metabolite families observed for each type of activity. The percentage represents the proportion of one activity compared to the whole occurrence of activities detected (n = 362). Some compounds present various activities and are considered several times.

Each of the 260 families of compounds was classified by chemical classes and bioactivity (Figure 3 and Figure 4). The 260 families of compounds were classified by their chemical classes, and 10 different classes were listed: alkaloids, depsipeptides, lipopeptides, macrolides/lactones, peptides, terpenes, polysaccharides, lipids, polyketides, and others (Figure 3). Of the 260 metabolite families, 66 belong to the peptide class. Together with the depsipeptide and lipopeptide classes, they represent 133 families of compounds (51%) derived from peptides. This is not surprising, regarding the diversity of biosynthetic pathways described in cyanobacteria: NRPS (non-ribosomal peptide synthase), PKS (polyketide synthase) and RiPPs (ribosomally synthesized and post-translationally modified peptides) with the ability to produce a wide range of metabolites and notable peptides [15] (Figure 3).

Fourteen major activities have been listed from the literature (lethality, neurotoxicity, hepatotoxicity, dermaltoxicity and cytotoxicity, anti-inflammatory, antioxidant, antiviral, anti-microalgal, antibacterial, antifungal, and antiprotozoal activities as well as protease and enzyme inhibition activities). Cytotoxic activity against various cell lines is the most frequently detected type of bioactivity with up to 110 families of the 260 listed. On the other hand, lethality and the antibacterial activities have been detected for 54 and 43 compound families, respectively (Figure 4).

**Figure 5:**
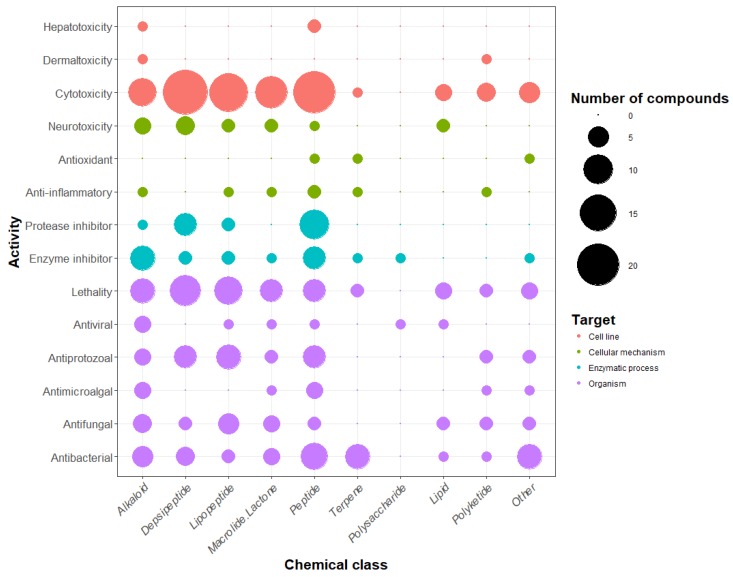
Classification of the 260 metabolite families according to their respective activities and chemical classes. The number of metabolite families is symbolized by the disc diameters, for each activity and each chemical class. For example, the first circle represents the number of alkaloids that exhibit a hepatotoxic activity (in this case, one family of metabolites). Colors correspond to the different categories of activity targets. For example, cytotoxicity and hepatotoxicity are tested in vitro against cell lines while neurotoxicity, antioxidant, and anti-inflammatory activities can be biochemically tested for specific cellular mechanisms (such as the sodium influx, the scavenging of ROS (reactive oxygen species), and the inhibition of cytokines).

The number of compounds displaying each tested activity is shown in Figure 5. The activities of molecules have been tested against different targets ranging from a specific cellular mechanism to an entire organism. For example, the inhibitory activity of proteases and other enzymes was shown to target enzymatic processes when the lethality and antimicrobial activity were tested against whole organisms. The lethality tests were generally realized against small invertebrates such as the brine shrimp crustacean *Artemia salina*, the gastropod mollusk *Biomphalaria glabrata*, and the crustacean *Thamnocephalus platyurus*. The present analysis confirms preceding observations (i.e., that cytotoxicity is the most commonly detected activity, followed by lethality and antibacterial activity). Some activities were detected only for a restricted number of compounds: dermaltoxicity concerned only two families of metabolites (aplysiatoxins and lyngbyatoxins) [51,52], hepatotoxicity was observed for three families (cylindrospermopsins, microcystins, and nodularins) [53,54,55], antioxidant and anti-inflammatory activities were observed for four (carotenoids, chlorophylls, mycosporine-like amino acids, and phycocyanins) [56,57,58,59], and seven metabolite families (coibacins, honaucins, aeruginosins, malyngamides, phycocyanin, scytonemin, and tolypodiol) [60,61,62,63,64,65,66], respectively. Nevertheless, there are only a few examples of these activities being tested by authors in comparison with cytotoxicity and lethality, which have been investigated far more regularly. In terms of anti-inflammatory activity, all seven tested molecules cited above were positive for this type of activity, and 53% of the studied molecule families have been tested for cytotoxic activity, while only 2.7% have been tested for anti-inflammatory activity. In parallel, some of these metabolite families can exhibit more than one activity. In fact, a total of 362 activities have been detected for the whole of the 260 metabolite families.

Focusing on the chemical classes, it appears that there is no specific indication that one chemical class exhibits specific activities with regard to other classes. The results from the review showed that the polysaccharide class presents only two tested activities (enzyme inhibition and antiviral activity), but only three types of polysaccharides isolated from cyanobacteria have been observed so far (calcium spirulan, cyclodextrins, and iminotetrasaccharide) [67,68,69]. Five chemical classes (the alkaloids, the depsipeptides, the lipopeptides, the macrolides, and the peptides) seem to present a remarkably large set of activities. When comparing the number of detected activities with the number of molecules belonging to each chemical class, the most bioactive molecules were shown to be the alkaloids, the lipopeptides, and the polyketides, which exhibit respectively 2.2, 1.9, and 1.8 activities per molecule on average.

These observations highlight a bias in the bioactivities searched from the isolated molecules. First, reported activities were those that researchers decided to test. Thus, the metabolite bioactivity profile could be underestimated because of the number of tests realized and remains the main limitation for the description of the potential applications of the bioactive molecules. In addition, there is still no consensus concerning the dose and dilution threshold that should be considered for each individual bioactivity test. In some cases, the concentration difference, used to determine if two distinct molecules are active, is important. For example, odoamide [70], which is a cyclic depsipeptide member of the aurilides family, and scytoscalarol [71], a sesterterpene, have both been described as being “cytotoxic.” However, their respective IC_50_ values appear to be very different: 26.3 nM against HeLa S3 human cervical cancer cells for odoamide and 135 µM against Vero cells for scytoscalarol, which represents a concentration difference of 500 times between their respective inhibition potentials. Furthermore, tests can be realized against several cell lines and strains presenting different sensitivity responses, which limit the comparison between results.

With 10 chemical classes and 14 types of bioactivity, the cyanobacterial metabolites are diverse and highly active. However, half of the families of metabolites listed in the database are peptides or peptide derivatives. This could be due to the importance of the peptide biosynthetic pathway (NRPS, PKS, and RiPPs) or the extraction methods used, which might favor peptide extraction. We did not observe a link between chemical classes and activities, but this observation must be considered carefully with regard to the low number of investigated molecules in some classes (i.e., polyketides, polysaccharides, and terpenes). The most frequently detected activity for cyanobacterial metabolites is cytotoxicity (42% of the metabolite families), whereas antioxidant or anti-inflammatory activities were detected for only 1.5% and 2.7% of the families. This imbalance is due to the frequency at which tests were carried out. In fact, cytotoxicity was tested for 53% of the molecules, while anti-inflammatory activity was only tested in 2.7%. This observation may reflect the research inclination to find new pharmaceutical compounds, notably cytotoxic compounds that are usable in cancer therapy, and suggests the potential for the discovery of new activities for application in other fields.

## 5. Beneficial Activities of Natural Products Produced by Cyanobacteria 

In this review, we further considered and developed 47 examples of molecules that are considered as exhibiting potential beneficial activities for several purposes. The 260 families of compounds could have a wide range of applications, e.g., agriculture, pharmacology, cosmetology, or in the food industry. For potential applications in agriculture, cyanobacterial compounds could be useful for alternative soil fertilization methods and as chemical pesticides [18]. The potential pharmaceutical applications of cyanobacterial metabolites include the development of new antibiotics, antibacterial drugs, or antiviral drugs [21]. Metabolite families were selected because of their specific features described below in each bioactivity-related section.

### 5.1. Antimicrobial Activity

Antimicrobial compounds that do not present toxic effects are particularly of interest for applications in the food industry in order to clean processing equipment or for food preservation [72,73]. Cyanobacteria produce 85 families of metabolites isolated from various strains, which display potent antimicrobial activity (representing a third of the 260 molecule families listed in the database) [18]. Below, we summarize the different antimicrobial metabolites (organized by type of antimicrobial activity) that have been isolated from cyanobacteria so far and the corresponding relevant information (see Table 1, Table 2, Table 3, Table 4 and Table 5). We also detail some examples of specific molecules that exhibit interesting bioactivity profiles such as the selective profile for their activity, which present broad-spectrum action together with the absence of associated cytotoxicity.

#### 5.1.1. Antibacterial Activity

Among the metabolite families listed, 43 molecules exhibit antibacterial activity, which represents 17% of the families. These components were, in general, tested against different types of bacteria: GRAM-negative, GRAM-positive mycobacteria, and cyanobacteria.

Among the 43 molecules, 22 are also cytotoxic and 16 present lethal activity against small invertebrates. Only three of them—eucapsitrione, kulolide-like molecules, and abietic acid—may have specific antimicrobial activity and no activity against other microorganisms.

Eucapsitrione and kulolide-like molecules (Table 1, details available in Supplementary Data S3) show antibacterial activity (against *Mycobacterium tuberculosis*) without inhibitory activity against the yeast *Candida albicans* [74,75]. Eucapsitrione is a anthraquinone derivative molecule isolated from the cyanobacterium *Eucapsis* sp. (UTEX 1519) [74]. This phenolic compound family is well-known in plants and some microorganisms, and has demonstrated a large range of bioactivities, including antimicrobial, antioxidant, anti-inflammatory, and potent anticancer properties [76,77,78,79]. This opens up other perspectives and applications for these anthraquinone derivatives isolated from cyanobacteria, such as eucapsitrione. However, so far, its other potential bioactivities have not been tested.

**Table 1:** Antibacterial molecules extracted from the database and discussed in this review.

Molecule Family	Chemical Classes	Activity	Producing Organisms	References
Eucapsitrione	Anthraquinone derivative	-Antibacterial -No antifungal -Cytotoxic	*Eucapsis* sp. UTEX 1519	[74]
Kulolide-like analogs	Depsipeptide	-Antibacterial -No antifungal -Antiprotozoal -Lethal -Cytotoxic -VGSC (Voltage Gate Sodium Channel) activation	*Lyngbya majuscula*, *Rivularia* sp., *Moorea producens*, *Okeania* sp., *Symploca hydnoides*, *Oscillatoria margaritifera*	[26,75,80,81,82,83,84,85,86,87,88,89,90]
Abietic acids	Terpene	-Antibacterial -No lethality -No antialgal	*Plectonema radiosum* LEGE 06105, *Nostoc* sp. LEGE 06077 and LEGE 07365, *Chroococcidiopsis* sp. LEGE 06174, *Synechocystis* sp. LEGE 06079, *Synechocystis salina* LEGE 06099, *Leptolyngbya ectocarpi* LEGE 11425, *Nodosilinea* sp. LEGE 13457, *Nodosilinea nodulosa* LEGE 07084	[91]
Hapalindole-like	Alkaloid	-Antibacterial -Antifungal -Antialgal -Cytotoxic -Insecticidal -Lethal activity -Reverse multidrug resistance (MDR) -VGSC modulator	*Hapalosiphon fontinalis*, *Westiellopsis* sp., *Fischerella musicola*, *Hapalosiphon welwitschii*, *Westiella intricata*, *Fischerella ambigua*, *Hapalosiphon delicatulus*, *Hapalosiphon hibernicus*, *Westiellopsis prolifica*, *Fischerella* sp., *Hapalosiphon laingii*	[92,93,94,95,96,97,98,99,100,101,102,103,104,105,106,107,108,109,110,111,112,113,114,115,116,117]

More details about compound activities are available in Supplementary Data S3.

**Table 2:** Antialgal molecules extracted from the database.

Molecule Family	Chemical Classes	Activity	Producing Organisms	References
Cyanobacterin	Lactone derivative	-Antialgal -Anti-cyanobacterial -Growth inhibition	*Scytonema hofmanni* UTEX 2349, *Nostoc linckia* CALU 892	[118,119,120,121]
Fischerellins	Polyketide	-Antialgal -Anti-cyanobacterial -Antifungal -Lethal -Growth inhibition	*Fischerella musicola*, *Fischerella* sp., *Fischerella ambigua*, *Fischerella tesserantii*	[122,123,124,125]
Westiellamide-like analogs	Peptide	-Antialgal -Anti-cyanobacterial -No antifungal -Lethal activity -Cytotoxic	*Westiellopsis prolifica* EN-3-1, *Nostoc* sp. 31, *Stigonema dendroideum* IA-45-3, *Oscillatoria raoi* TAU IL-76-1-2, *Nostoc spongiaeforme* var. *tenue* str. Carmeli	[126,127,128,129,130,131]
Ambigols	Alkaloid	-Antialgal -Antibacterial -Antifungal -Antiprotozoal -Lethal activity -Cytotoxicity -Enzyme inhibition	*Fischerella ambigua* 108b	[132,133]
Schizotrin-like analogs	Peptide	-Antialgal -Antibacterial -Antifungal -Antiprotozoal -Lethal activity -Cytotoxicity	*Schizothrix* sp. TAU IL-82-2, *Lyngbya* sp. 36.91, *Phormidium* sp. LEGE 05292, *Tychonema* sp. CCAP 1462/13	[134,135,136,137,138,139,140,141]

The kulolide-like family includes 44 related molecules. Kulolide, which is the first molecule of the family to be discovered, was isolated from a cephalaspidean mollusk *Philinopsis speciosa* [80]. Luesch and co-workers (2001) isolated the first cyanobacterial analogues of this family, naming them the pitipeptolides, and proposed a cyanobacterial origin for kulolide itself [89]. All members of the kulolide-like family share chemical similarities and can be categorized into two subgroups: those containing 2,2-dimethyl-3-hydroxy-7-octynoic acid (Dhoya) and those containing 3-hydroxy-2-methyl-7-octynoid acid (Hmoya) [26]. The same activities were not tested for all analogues, but some of them have shown antibacterial, antiprotozoal, cytotoxic, and even lethal activities (Table 1).

The third example of a family of molecule presenting a specific anti-bacterial activity is that of abietic acids (Table 1). Abietic acid is a terpene that is generally found in resin and used by conifers as a defense metabolite [91]. It demonstrates anti-cyanobacterial activity against *Synechococcus nidulans*, and seems to be non-toxic to *Chlorella vulgaris* and the brine shrimp *Artemia salina* (Table 1). It has been suggested that its activity and defense mechanisms could be equivalent to those of coniferous plants, i.e., trapping microorganisms or acting as allelochemical compounds. These non-toxic properties are compelling for the development of specific anti-cyanobacterial products.

The hapalindole-like group is a family of alkaloids, which contains around 80 related molecules [92,93,94,95,96,97,98,99,100,101,102,103,104,105,106,107,108,109,110,111,112,113,114,115,116,117] (Table 1). These metabolites were previously isolated from *Hapalosiphon*, *Fischerella*, *Westiellopsis*, and *Westiella* genera. They show a wide range of activity, most notably, antibacterial activity against 27 various bacterial strains, together with antifungal and antialgal activities. They are also cytotoxic and exhibit additional insecticidal activity. Some of them were even able to reverse drug resistance in cancer cell lines [97,116] (Table 1). They putatively exhibit modulatory activity on sodium channels [95], which could explain their diverse bio-activities.

#### 5.1.2. Antialgal Activity

Antialgal activity was tested generally against microalgae, and 10 families of metabolites were shown to present such activity. Among these 10 families, four also exhibited anti-cyanobacterial activity, and it can be supposed that these molecules may be acting against general photosynthesis mechanisms. For example, cyanobacterins isolated from two strains, *Scytonema hofmanni* UTEX 2349 and *Nostoc linckia* CALU 892 [118,119], were shown to present significant antimicrobial activity directed against a large panel of microalgal and cyanobacterial strains (Table 2). These compounds also inhibit the growth of eight angiosperm plants, such as duckweed (*Lemna* genus), pea, corn, sorrel, black bindweed, wild oat, and green foxtail [120] (Table 2). Gleason and Case (1986) showed that this activity is due to the inhibition of the Hill reaction in photosystem II without inhibition of photosystem I [120].

Another example is the fischerellin family. These compounds were observed in four strains belonging to the *Fischerella* genus. They show a wide range of activities comprising growth inhibition of *Lemna minor*, antifungal and lethal activities, and antialgal and anti-cyanobacterial activities. Hagmann & Jüttner (1996) showed that fischerellins A is an effective inhibitor of photosystem II [123] (Table 2).

The westiellamide-like analogs family comprise 12 related cyclic peptides isolated from five strains belonging to four different genera (Table 2). The related molecules, known as the bistratamides, were previously isolated from the ascidian *Lissoclinum bistratum* [142], and authors hypothesized a cyanobacterial symbiont origin for these molecules [128]. This family of compounds have been shown to have anti-algal and anti-cyanobacterial activities (Table 2), but they did not show any antifungal activity against the yeast *Saccharomyces cerevisiae* [126,127,128,129,130,131]. Moreover, one of them, dendroamide A, has shown the ability to reverse the multi-drug resistance of a human breast carcinoma cell line (MCF-7/ADR) [126]. The MCF-7/ACR cell line overexpresses the P-glycoprotein pump, which transports drugs outside of the cell, providing higher resistance to chemical treatment. Dendroamide A is able to specifically inhibit the action of the P-glycoprotein pump, which allows the drug to penetrate and lyse the cells, so it has potential anticancer applications.

Among the antialgal compounds, two have a remarkably broad spectrum of antimicrobial activities: the ambigols and the schizotrin-like analogs families, both show antialgal, antibacterial, antifungal, and antiprotozoal activities (Table 2). Three ambigol variants were isolated from *Fischerella ambigua* strain 108b, while the schizotrin-like family includes 13 structurally related molecules isolated from four different strains (Table 2). In addition to these antimicrobial activities, the ambigols also present enzyme inhibition activity against cyclooxygenases and HIV-1 reverse transcriptase. The members of the schizotrin-like family, the portoamides (isolated from *Phormidium* sp. LEGE 05292), have also shown mitochondrial metabolism inhibition activity, which induces a further decrease in the cellular ATP content in cells exposed to portoamides [140]. This property is also promising for the development of drugs acting against tumors and cancers [143].

Via their main antialgal action (i.e., photosynthesis inhibition), the molecules have been shown to present other potential uses and could be used as alternatives to chemical herbicides based on PSII inhibition (e.g., 3-(3,4-dichlorophenyl)-1,1-dimethylurea, DCMU). These families of compounds could be used to develop new algaecides and herbicides and/or to develop new pharmaceutical drugs.

**Table 3:** Antifungal molecules extracted from the database.

Molecule Family	Chemical Classes	Activity	Producing Organisms	References
Hassallidins	Glycolipopeptide	-Antifungal -No antibacterial activity	*Hassalia* sp. B02-07, *Anabaena* sp. (SYKE 748A, 90y1998, 90M3, 299B, 258, SYKE763A, 0TU33S16, 0TU43S8, 1TU33S8, 1TU35S12, 1TU44S9, 1TU44S16, SYKE971/6, NIVA-CYA269/2, NIVA-CYA269/6, XPORK5C, XSPORK7B, XSPORK36B, XSPORK14D, BECID19), *Anabaena cylindrica* Bio33 *Cylindrospermopsis raciborskii* (ATC-9502 & CS-505), *Aphanizomenon gracile* Heaney/Camb 1986 140 1/1, *Nostoc* sp. (159 & 113.5), *Tolypothrix* sp. PCC 9009 *Planktothix serta* PCC 8927	[144,145,146,147,148]
Lyngbyabellins	Depsipeptide	-Antifungal -No antibacterial activity -Lethal activity -Cytotoxic	*Lyngbya majuscula* *Lyngbya* sp., *Lyngbya bouillonii* *Moorea bouillonii*	[149,150,151,152,153,154,155,156]
Micro-guanidines	Guanidine derivative	-Antifungal -No cytotoxicity -No protease inhibition	*Microcystis* sp. TAU IL-306, *Microcystis aeruginosa* TAU IL-374	[157,158,159]
Majusculamides	Lipopeptide	-Antifungal -Cytotoxic -Immunosuppressive activity -Actin filaments disrupting -Anti-settlement activity	*Lyngbya majuscula*, *Lyngbya polychroa*	[160,161,162,163,164,165,166]

#### 5.1.3. Antifungal Activity

Twenty-eight families of compounds showed antifungal activities. Toxicity tests were carried out against diverse fungal species, which are mostly pathogenic ones. Quite common ones include *Candida albicans*, *Saccharomyces cerevisiae*, *Penicillium notatum*, and *Aspergillus oryzae*, and less common ones include *Trichophyton mentagrophytes* and *Ustilago violacea*. Among these compounds, 11 showed several other types of antimicrobial activity in addition to antifungal activity. Only two metabolite families, hassallidins and lyngbyabellins, demonstrated specific antifungal activity without presenting any antibacterial activity. The hassallidins are cyclic glycolipopeptides isolated from three strains belonging to the Nostocales (Table 3). Four variants have been characterized so far [144,145,146,147,148], and the non-ribosomal peptide gene cluster responsible for hassallidin biosynthesis has been identified. Thus, the hassallidins cluster was detected by bioinformatics analysis of the genomes of four heterocytous cyanobacteria, *Aphanizomenon gracile*, *Cylindrospermopsis raciborskii*, *Nostoc* sp., and *Tolypothrix* sp., and hassallidins production was confirmed by LC/MS analysis (Table 3). Recently, Pancrace et al. (2017) identified the hassallidins gene cluster and characterized a new hassallidins variant from *Planktothrix serta* (PCC 8927), which is a nitrogen-fixing, non-heterocytous forming strain [147]. They concluded that the strain gain of the cluster occurred by horizontal transfer and, therefore, questioned the natural product distribution and diversity among cyanobacteria.

The lyngbyabellins are cyclic depsipeptides. They were isolated from *Lyngbya* and *Moorea* species (Table 3). Hectochlorin is the only member of the family that was tested for antibacterial and antifungal activity, which showed no antibacterial activity but displayed antifungal activity against *Candida albicans* [152]. The distinctive feature of the lyngbyabellins is that they can also disrupt actin filaments. Luesch et al. (2000) [151] and Han et al. (2005) [150] showed that cells exposed to Lyngbyabellin A and E lost their microfilament network, which caused cell cycle arrest at the cytokinesis phase. Marquez et al. (2002) [152] showed that the same process appears with cells exposed to hectochlorin. They also demonstrated that the molecule stimulates actin polymerization and then induces cell cycle disorders.

Microguanidines are guanidine derivatives isolated from two strains of *Microcystis* (Table 3). These molecules showed antifungal activity against *Saccharomyces cerevisiae* E4orf4 without cytotoxic activity. This specificity could be of interest for the development of new antifungal products [158].

Majusculamides are lipopeptides produced by *Lyngbya majuscula* and *Lyngbya polychroa*. These metabolites combine antifungal and cytotoxic activities with immunosuppressive and anti-settlement properties [160,161,162,163,164,165,166]. Simmons et al. (2009) [165] also demonstrated the ability of majusculamides to disrupt actin filaments that may explain these specific properties (Table 3).

#### 5.1.4. Antiviral Activity

Viral diseases are one of the main health concerns around the world. According to the World Health Organization (WHO), HIV and AIDS caused around one million deaths in 2017 [167]. We noted that eight families of cyanobacterial compounds have shown antiviral activity. Antiviral activity was generally determined by testing against the human immunodeficiency virus (HIV-1 or HIV-2) or the *Herpes simplex virus* (HSV-1 or HSV-2). The aplysiatoxins showed activity against Chikungunya’s virus (CHIKV) [168] (Table 4), but are also very active dermatotoxins [51,169] and tumor-promoting molecules due to their capacity to activate protein kinase C (PKC), which is an enzyme that plays roles in cell proliferation, differentiation, and apoptosis [168] (Table 4). Recently, Han et al., demonstrated that two aplysiatoxin analogues showed the capability to inhibit the potassium channels [170], which opens interesting perspectives for the study and use of these molecules for drug development.

**Table 4:** Antiviral molecules extracted from the database.

Molecule Family	Chemical Classes	Activity	Producing Organisms	References
Aplysiatoxins	Alkaloid	-Antiviral -Dermatitis and swimmer’s itch agents -Cytotoxic	*Lyngbya majuscula*, *Schizothrix calcicola*, *Oscillatoria nigro-viridis*, *Trichodesmium erythaeum*	[51,168,170,171,172,173]
Cyanovirin-N	Protein	-Antiviral -No cytotoxicity -Stop fusion and transmission of HIV-1 virus	*Nostoc ellipsosporum* *Cyanothece* sp.	[174,175,176]
Calcium spirulan	Polysaccharide	-Antiviral -No cytotoxicity -Low anticoagulant activity	*Arthrospira platensis*	[67,177,178]

Two other families of molecules have shown antiviral activity against a large panel of viruses. The first one, cyanovirin-N analogs, have been isolated from *Nostoc ellipsosporum* [174] and *Cyanothece* sp. [176] (Table 4). These molecules are proteins belonging to the lectins class because of their ability to bind glycans. Cyanovirins show inhibitory activity against HIV-1, HIV-2, simian immunodeficiency virus (SIV), feline immunodeficiency virus, HHV-6, and measles virus [174,175]. Also, they inhibit Ebola and influenza viruses [176]. Nevertheless, cyanovirins are not active against some viruses, such as human herpesvirus A (HHV-1), cytomegalovirus, and adenovirus type 5 [175]. Cyanovirins are also non-cytotoxic for non-infected cells (at concentrations required for antiviral activity) [174,175] (Table 4). In fact, cyanovirin-N binds gp120, which is a glycoprotein component of the HIV envelope. As a result, the molecule inhibits membrane fusion into target cells and stops virus transmission. Calcium spirulan has been isolated from *Arthrospira platensis* (anc. *Spirulina platensis*) and is a sulphated polysaccharide. It shows antiviral activity against a wide range of viruses including HIV-1, HSV-1, the human cytomegalovirus (HCMV), measles virus, mumps virus, and influenza virus, in addition to a low cytotoxicity against several cell lines (ID50 values between 2900 and 7900 µg/mL) (Table 4) [67,178]. Furthermore, calcium spirulan seems inactive against *Poliovirus* and *Coxsackievirus*, two non-enveloped viruses, which means that it likely has selective activity for enveloped viruses. Hayashi et al. (1996) [67] also showed that this molecule inhibits virus penetration in targeted cells. Other sulphated polysaccharides are known for their anticoagulant and antiviral activity, such as heparin or dextran sulphate [179,180]. In comparison to these molecules, calcium spirulan showed a lower anticoagulant activity and a longer half-life in blood [177], which confirms its promising potential for the development of new specific antiviral drugs.

#### 5.1.5. Antiprotozoal Activity (Against Malaria, Leishmaniosis, Chagas Disease)

The last kind of antimicrobial properties tabulated is antiprotozoal activity. Protozoans are eukaryotic microorganisms, some of them have parasitic lifestyles and are well-known for their involvement in human diseases such as Malaria, Leishmaniosis, Chagas’ disease, and Trypanosomiasis. These diseases represent a huge problem in tropical countries where the parasite is transmitted by mosquitoes. The WHO identified more than 210 million Malaria cases in 2016 [181]. Therein, molecules with antiprotozoal activity are actively being sought in order to develop new drugs against these diseases.

**Table 5:** Antiprotozoal molecules extracted from the database.

Molecule Family	Chemical Classes	Activity	Producing Organisms	References
Companeramides	Depsipeptide	-Antiprotozoal -No significant cytotoxicity	*Leptolyngbya* sp. or «*Hyalidium*»	[29]
Hoshinolactam	Lactam	-Antiprotozoal -No cytotoxicity	*Oscillatoria* sp.	[182]
Dolastatins	Peptide	-Antiprotozoal -Lethal -Cytotoxic	*Lyngbya majuscula*, *Symploca hydnoides*, *Lyngbya* sp., *Symploca* sp. VP642, *Lyngbya-Schizothrix* assemblage	[30,32,75,183,184,185,186,187,188,189]

From the review, 28 cyanobacterial metabolites showed antiprotozoal activities. Tests have been conducted against several strains of *Plasmodium falciparum* (causative agent of Malaria), *Leishamania donovani* (Leishmaniosis), *Trypanosoma cruzi* (Chagas’ disease), and *Trypanosoma brucei* (Sleeping sickness). Among the 28 concerned families of molecules, 19 showed antiprotozoal activity against drug-resistant strains, especially against chloroquine-resistant strains of *Plasmodium falciparum* (see Table 5). Nevertheless, most of them are less active than the antibiotics currently used. For example, companeramides are cyclic depsipeptides produced by a cyanobacterium previously identified as *Leptolyngbya* sp. (now *Hyalidium*) [29] (Table 5). Companeramides showed antimalarial activity against three strains of chloroquine-resistant *Plasmodium falciparum*. They also showed no significant cytotoxicity against the cell lines used in the test, which constitutes a unique property for the development of specific but non-toxic antimalarial drugs. Unfortunately, the activity of companeramides against the parasite is 100-fold lower than that of chloroquine (a commonly used drug), which reduces their potential utilization.

However, some molecules show promise as substitutes for antibiotic treatment because of their strong activity against the parasite. This is the case for hoshinolactam and dolastatins. Hoshinolactam is an aromatic molecule belonging to the lactam chemical class [182]. It was isolated from an environmental sample rich in *Oscillatoria* sp. and has shown antiprotozoal activity against *Trypanosoma brucei* (IC_50_ = 3.9 nM) with no cytotoxicity against MDR-5 (the host cell, IC_50_ > 25 µM) (Table 5). Furthermore, the IC_50_ of pentamidine (another commonly used drug) against *Trypanosoma* species is 4.7 nM. Thus, the activity of hoshinolactam is equivalent to that of the antibiotics, and hoshinolactam represents a promising alternative to pentamidine for Trypanosomiasis treatment [182].

Dolastatins are a well-studied family of peptides. The first members of this family were isolated in 1977 from the sea hare *Dolabella auricularia* [190]. In 1998, other molecules belonging to the dolastatins family were isolated from the cyanobacteria *Lyngbya majuscula* and *Symploca hydnoides*, which leads to the hypothesis that dolastatins isolated from the mollusc have a cyanobacterial dietary origin [191]. Dolastin 10, which is one of the dolastatin-related molecules, is the most potent antiprotozoal metabolite discovered so far from cyanobacteria. This exhibits an IC_50_ of 0.1 nM (the IC_50_ of chloroquine is, on average, 5 nM for the chloroquine-sensitive strain of *P. falciparum*) [184]. Dolastatins are also strongly cytotoxic molecules (Table 5). They are able to inhibit tubulin polymerization, which induces cellular cycle arrest and apoptosis [192]. Antiprotozoal and cytotoxic activities are both the result of this property. Therefore, there is no apparent specificity for this molecule to act directly against the parasite itself, where the cellular host is likely the most potent target of dolastatins. For this reason, Fennel et al. (2003) [184] concluded that dolastatins do not constitute a promising antiprotozoal drug despite their strong activity.

### 5.2. Potential Anticancer Activity

Currently, cancers constitute the most important non-transmittable diseases worldwide. According to the WHO, cancer was the cause of one in six deaths (9.6 million) in 2018 [193]. The annual cost of cancer in 2010 was estimated to be $1.16 trillion USD [194]. That is why numerous studies have been conducted to understand the physiology of different cancers and to find new efficient anticancer drugs. For this purpose, researchers are looking for molecules, and, notably, natural products that are able to kill cells or inhibit cell proliferation. In this section, we selected some particular cyanobacterial metabolites for which the mode of action is known as significant examples. We feature metabolites acting on the microtubule or the microfilament network, histone deacetylase and proteases inhibitors, and molecules with the ability to reverse multi-drug resistance.

#### 5.2.1. Cytotoxic Activity

The first type of activity test was performed to determine the potential of molecules as anticancer agents due to cytotoxicity. Different cell lines derived from tumor cells, like the HeLa cell line (derived from cervical cancer), KB (HeLa derivative), LoVo (human colon tumor), H-460 (human lung cancer), and MCF-7 (human breast cancer) have been used to assess this activity. Most of the time, the molecules investigated were tested against two or more cell lines to detect a potent specificity and to evaluate their potential for drug development. According to this review, 110 families of metabolites isolated from cyanobacteria showed cytotoxicity, which represents 43% of the molecule families listed in the database.

The best example of potent anticancer molecules derived from cyanobacteria is the dolastatin family [191]. One synthetic analogue of dolastatin 10, monomethyl auristatin E, is actually used to treat Hodgkin’s lymphoma in the drug Brentuximab vedotin [191]. Luesch et al. (2001) [187] showed that dolastatin 10 and symplostatin 1 are 100-fold more efficient than vinblastine (anticancer drug extracted originally from the Madagascar periwinkle) against the same cell line due to their ability to depolymerize microtubules. Unfortunately, dolastatins also have strong cytotoxicity [187,195]. Researchers found a way to reduce this toxicity by coupling monomethyl auristatin E with a chimeric antibody against CD30 (tumor necrosis factor receptor, highly expressed in Hodgkin’s lymphoma) in order to target only tumor cells [196]. Since then, other antibody drugs linked (ADC) with monomethyl auristatin E have been developed. For example, glembatumumab vedotin is currently under clinical trial. This drug targets GPNMB (glyprotein non-metastatic melanoma protein B), which is a glycoprotein expressed in melanoma and breast tumors [197]. In addition to the dolastatins, other cyanobacterial metabolites destabilize the microtubule network. Notably, one such metabolite is tubercidin, which is a nucleoside produced by *Tolypothrix byssoidea*, *Tolypothrix distorta*, *Plectonema radiosum*, and *Scytonema saleyeriense* var. *indica* [198,199]. This molecule was previously isolated from the bacterium *Streptomyces tubercidicus*. Tubercidin has shown inhibition of cell proliferation with an IC_50_ of 248 nM (Table 6). Tubercidin acts against dolastatins showing a microtubule stabilizing activity comparable to taxol bioactivity [200]. Its cytotoxicity is due to its stabilizing property, which causes mitotic arrest at G2/M transition and stops growth [201].

Another mechanism of cytotoxicity noted from cyanobacterial metabolites is the destabilization of actin microfilaments. As tubulin microtubules, actin microfilaments are key cytoskeleton components of cells. Microfilaments are involved in several mechanisms: cell division (cytokinesis), cell motility, cell adhesion, exocytosis, and endocytosis [211]. Thus, molecules with actin-modulating activity are sought in order to develop anticancer drugs because of their ability to induce apoptosis [211]. Four cyanobacterial metabolite families have shown disrupting activity of the actin microfilament network: the lyngbyabellins, the majusculamides, the aurilides, and the swinholide-like molecules (Table 6). 

Lyngbyabellins and majusculamides, as mentioned above, have shown antifungal activity that likely corresponds to their ability to modulate actin polymerization [150,151,152,165]. Aurilides are cyclic depsipeptides, and the first member of this family was isolated from the sea hare *Dolabella auricularia* [212]. Since then, seven other related molecules have been isolated from two cyanobacterial genera: *Lyngbya* and *Okeania* [70,202,203,204,205], and one from *Philinopsis speciosa* (cephalaspidean mollusc) [213]. Aurilides showed nanomolar cytotoxic activity associated with a moderate toxicity to *Artemia salina*. Two analogues, lagunamides A and B, have also shown antimalarial activity and anti-swarming activity against *Pseudomonas aeruginosa* [203] (Table 6). Han et al. (2006) [202] showed that aurilides induce microfilament disruption at the micromolar level. They concluded that this disrupting activity is likely related to their toxic and antimicrobial activities. 

Swinholide-type molecules were macrolides, originally isolated from the sponge *Theonella swinhoei* [214]. In 2005, Andrianasolo et al. (2005) [206] succeeded in isolating swinholide A and two new related molecules (ankaraholides A and B) from two cyanobacteria (*Symploca* sp. and *Geitlerinema* sp., respectively), which leads to the hypothesis of a symbiotic origin of the compounds isolated from the sponge [206] (Table 6). More recently, Humisto et al. (2018) identified the swinholide biosynthetic cluster in *Nostoc* sp. (Table 6) [207], and Tao et al. (2018) isolated nine swinholide-related metabolites from a marine *Phormidium* sp. [208]. Swinholide A, isolated from the marine sponge, showed microfilament-disrupting activity by stabilizing actin dimers [215]. In addition to their cytotoxic activity, cyanobacterial swinholides also showed the same actin-disrupting activity, which is of interest for the development of related anticancer drugs [206]. 

Other metabolites with noticeable cytotoxicity are anabaenolysins, which are lipopeptides isolated from two strains of the *Anabaena* genus [210] (Table 6). Anabaenolysins showed cytotoxicity against all of the 10 cell lines tested, with LC_50_ values between 4 and 20 µM depending on the cell lines and the anabaenolysin variants [210]. In addition, using a trypan dye exclusion assay, these authors showed that anabaenolysins have a unique profile. Instead of excluding the dye, cells showed an influx of trypan dye, which means that anabaenolysins permeabilize cell membranes until necrotic death [210]. Anabaenolysins are able to solubilize the lipid component of the cell membrane, and likely acts with the same mechanism as the detergent digitonin. Anabaenolysins particularly target cholesterol-containing membranes and do not induce permeabilization of mitochondria membranes. As detergents, anabaenolysins also show hemolytic activity, but at lower concentrations than digitonin and surfactin [209]. In addition, Oftedal et al. (2012) showed that the permeabilization ability of anabaenolysins also allows the internalization of nodularin [209]. This property is of interest for the development of a drug administration strategy involving anabaenolysins as a synergistic compound and other bioactive molecules that cannot be passed through the membrane within the targeted cells alone.

Six cyanobacterial families of compounds showed the ability to reverse multi-drug resistance (MDR) in addition to their cytotoxic properties. These include the cryptophycins [216,217,218,219], the hapalindole-like metabolites [116], hapalosin [220], the patellamides [221,222], the tolyporphins [223,224], and the westiellamide-like [126] molecules (see Supplementary Data S3). Among them, five families displayed MDR reversal activity by acting on the P-glycoprotein pumps (except for cryptophycins and patellamides for which the MDR reversal mechanisms have still not been described). P-glycoprotein is a glycosylated transmembrane protein that transports drugs and toxins out of the cell. This protein is often overexpressed in cancer cells and leads to resistance against standard chemotherapeutics because of its lower accumulation in targeted cells [225]. Thus, metabolites with the ability to inhibit this efflux pump are of interest for developing anticancer drugs or to supplement current chemotherapeutic strategies in order to increase their efficiency on resistant cancer cells.

#### 5.2.2. Protease Inhibitory Activity

Proteases are a widespread family of enzymes found in most, if not all, organisms. They are involved in a large number of pathways including coagulation, inflammation, digestion, hemostasis, and blood pressure regulation [226,227]. There are several types of proteases that are classified by their specific hydrolysis mechanisms. The major groups are the metalloproteinases, the serine proteases, the cysteine proteases, the threonine proteases, and the aspartic acid proteases [227]. Because of their ubiquity, these enzymes are attractive targets for the development of new drugs against diverse diseases [226]. Some proteases have also shown the potential to act against thrombotic diseases [226], hypertension [227], pulmonary diseases [228], asthma [229], pathogenic microorganisms [230,231], and even cancers [227,232]. According to our investigation, 24 families of metabolites presenting diverse protease inhibitor activities have been isolated from cyanobacteria to date. These compounds have shown inhibitory activity against a wide range of proteases, including enzymes belonging to the cathepsin family or the well-known serine proteases trypsin, chymotrypsin, and thrombin. Only three metabolite families have shown an inhibitory activity against cathepsins. Cathepsins are frequently overexpressed in cancer cells and are involved in tumorigenesis, cell invasion, and metastasis [233,234,235,236,237,238]. One of these three families, the spumigins, isolated from *Nodularia spumigena* and *Anabaena compacta* [239,240,241], is a set of linear peptides that are structurally similar to the aeruginosins (Table 7). They showed inhibitory activity against several proteases including trypsin, thrombin, plasmin, and cathepsin B [240]. All of these proteases are potentially involved in cancer cell processes, and, notably, cathepsin B has been proposed to be a promising target for anticancer drug development [234,242].

Another example of metabolites with interesting activity is the cyanopeptolin-like family. This family is the second in terms of the number of structural analogues isolated, after the microcystins (respectively 140 and 246 molecular variants described so far). Currently, more than 50 papers have reported on the isolation and activities of these metabolites. They are cyclic depsipeptides isolated from 12 different cyanobacterial genera (Table 7). Among the large number of analogs, a wide range of activity has been reported for these cyanobacterial metabolites including protease activity and other types of enzyme inhibition, cytotoxicity, lethal activity, and antimicrobial activity, which open various possibilities for developing therapies targeting cancer cells or microorganisms or those that fight some diseases like emphysema [277], pancreatitis [295], or thrombosis [296]. Nevertheless, this large number of activities can also represent a problem such as how to develop a therapeutic drug exhibiting a specific activity. It would be interesting to study some analogs in more depth or to conduct a structure–activity relationship study in order to increase the specificity of synthetic variants.

Lastly, another class of inhibitors that would be of interest for the development of new therapeutics against tumors is the proteasome inhibitors. Proteasome or ubiquitin-proteasome is a multi-enzymatic complex of eukaryotes. It is involved in protein degradation in a different way than the lysosomes [296]. Because proteasome catalysis is involved in a wide variety of essential pathways, including cell-cycle progression and the regulation of apoptosis, it is a potent target for cancer therapy. Moreover, malignant cells have been shown to be more affected by proteasome inhibitors than normal cells, which reduce the potentially deleterious side effects of these molecules [232]. Four cyanobacterial families of metabolites were described to inhibit the 20S core of proteasome: the carmaphycins [293], the cylindrocyclophanes [169,297,298,299,300,301,302,303], the nostocyclopeptides [304,305], and nostodione [306,307] (see Supplementary Data S3). Among them, the most efficient 20S proteasome inhibitors are the carmaphycins, which exhibit an IC_50_ of around 2.5 nM [293], whereas the other compounds present a micromolar range of action [169,305,306] (Table 7). Only two carmaphycin variants (A and B) have been isolated from *Symploca* sp., so far. These molecules are linear peptides with cytotoxic and antiprotozoal activities. They show the additional ability to inhibit the 20S proteasome activity in yeast and *Plasmodium* by interacting with the β5 subunit [293,294]. These bioactivities are interesting for the use of carmaphycins as anticancer or antimalarial therapeutics. Two studies were conducted to enhance the specificity of carmaphycins for either applications. To develop a specific antimalarial drug, LaMonte et al. (2017) synthesized synthetic analogues of carmaphycin B and identified one analog with a selectivity index of 380 for antiprotozoal activity over cytotoxic activity [294]. On the other hand, Almaliti et al. (2018) studied the potential of carmaphycins as anticancer drugs and as an antibody–drug conjugate (ADC) in order to enhance the selectivity of the molecules for cancer cells and to reduce the potential side effects [308]. 

Therefore, cyanobacterial metabolites with protease inhibition activities were shown to be less specific for further use, but the synthesis of synthetic analogs increased the selectivity of some of these molecules.

#### 5.2.3. Histone Deacetylase Inhibitors

Histone deacetylases (HDACs) are enzymes involved in re-modeling the chromatin and the acetylation/deacetylation of histone and non-histone proteins. Furthermore, histone deacetylases play a key role in histone–DNA interactions and in the binding to transcription factors. HDACs have also been identified as potent regulators of gene expression [309,310]. Because cancer generally emerges from genetic mutations inducing hyperactivation of oncogenes or loss of tumor-suppressor genes, targeting mechanisms that are involved in the epigenetic regulation of genes is a promising strategy for the development of anti-tumor drugs [310].

Two molecules showing histone deacetylase inhibitory activity have been isolated from cyanobacteria so far, which are known as largazole and santacruzamate A. Both of these molecules come from *Symploca* sp. strains (Table 8). Largazole has shown inhibition against 12 class I HDACs in addition to inhibition of the ubiquitin-activating enzyme (E1). It has also shown cytotoxicity to several cell lines (Table 8). Largazole acts as a pro-drug—the molecule needed to be activated by hydrolysis to release its active form, the largazole thiol [309]. Santacruzamate A has also shown histone deacetylase inhibition and cytotoxicity. It shares some structural features with suberoylanilide hydroxamic acid (SAHA), which is a clinically approved HDAC inhibitor that is used to treat refractory cutaneous T-cell lymphoma [317]. Salvador-Reyes and Luesch (2015) performed an in-depth review of the activities and mechanisms of action of these two metabolites [309]. They highlighted the high potency of largazole in anticancer drug development, while the potency of santacruzamate seems to remain more limited.

### 5.3. Anti-Inflammatory and Antioxidant Activity

In this section, we specifically describe cyanobacterial metabolites that showed no cytotoxicity associated with their anti-inflammatory or antioxidant properties.

#### 5.3.1. Anti-Inflammatory Activity

According to our review, seven metabolite families isolated from cyanobacteria were found to have anti-inflammatory activity (aeruginosins, coibacins, honaucins, malyngamides, phycocyanin, scytonemin, and tolypodiol). 

Currently, anti-inflammatory molecules have been widely studied in order to develop new therapeutics directed against chronic inflammatory diseases, such as rheumatoid arthritis, psoriasis, chronic obstructive pulmonary disease, multiple sclerosis, and inflammatory bowel disease [318]. Anti-inflammatory compounds can also be useful against cardiovascular diseases, such as atherosclerosis [319], and neurodegenerative diseases like Parkinson’s disease [320].

Anti-inflammatory tests have been performed in vitro or in vivo in mice. For example, malyngamides have been shown to inhibit superoxide production generated by inflammation-promoting agents [332], and honaucins inhibit pro-inflammatory cytokine expression [61] in the murine macrophage cell line RAW264.7. The mouse ear edema assay has been performed in vivo by observing the resorption of ear edema in the presence of anti-inflammatory compounds, such as phycocyanin [64], scytonemin [330], and tolypodiol [66], which have shown noteworthy activities by using this assay.

Three metabolites seem to be particularly interesting according to their specific bioactivity profiles: the aeruginosins, phycocyanin, and scytonemin, which have not shown any toxicity when tested in vitro or in vivo. Aeruginosins have shown anti-inflammatory properties using the AlphaLISA assay. They are able to down-regulate the level of pro-inflammatory mediators (IL-8 and ICAM-1) in stimulated endothelial cells [62] without affecting the viability of two different cell lines [62] (Table 9). Aeruginosins have also shown serine protease inhibitory activity against trypsin, thrombin, and plasmin [321], and their corresponding biosynthetic gene cluster was first identified in *Planktothrix agardhii* and *Nodularia spumigena* (Table 9) [241,322]. Currently, no correlation between serine protease inhibition and the anti-inflammatory activity of aeruginosins were shown. However, on neutrophils, it has been shown that some serine proteases (elastase, cathepsin G, and proteinase 3) are responsible for the conversion and activation of proinflammatory chemokines (and notably, interleukine-8 (IL-8)) and are able to conserve or enhance the inflammation response [333,334,335]. In this regard, it will be compelling to further test whether aeruginosins are capable of inhibiting other serine proteases, notably elastase, cathepsin G, and proteinase 3, in order to determine whether the down-regulation of IL-8 induced by the aeruginosins is mediated through serine protease inhibition processes.

Phycocyanin is a phycobiliprotein, constituting one of the major cyanobacterial pigments, together with the chlorophylls and phycoerythrin. It is involved in light-harvesting and the energy transfer of phycobilisomes within the outer membrane of thylakoids. In addition, phycocyanin has shown a wide variety of beneficial properties including antioxidant, anti-inflammatory, neuroprotective, and hepatoprotective activities [64] (Table 9). Authors of phycocyanin studies have reviewed the main features of phycocyanin anti-inflammatory mechanisms. Phycocyanin is able to scavenge ROS, has anti-lipoperoxidative effects, and inhibits cyclooxygenases (specifically COX-2) as well as TNF-α release. All of these properties are unique from the perspective of new therapeutics development targeting neurodegenerative diseases such as Alzheimer’s disease, Parkinson’s disease, or Huntington’s disorder, or as an anti-inflammatory agent [64].

Scytonemin is an alkaloid pigment found in the sheath of some cyanobacteria and, particularly, on some organisms living in extreme environments [65]. Scytonemin synthesis is mainly induced by UV-A exposure in order to reduce heating and the oxidative stress [65]. Scytonemin is mainly involved in photoprotection by UV-absorption [65]. It has also been shown to have anti-inflammatory activity with no cytotoxicity against non-proliferating cells [65,330,331]. In addition, scytonemin has been shown to inhibit polo-like kinase 1 (PLK1), which is an enzyme involved in the phosphorylation and activation of proteins such as cdc25C, which is involved in cell cycle progression and the G2/M transition in the cell cycle (Table 9). As a consequence, scytonemin can repress cell proliferation [65,330,331]. Therefore, scytonemin could be a promising compound for use in the development of anticancer therapeutics, sunscreen agents, or anti-inflammatory drugs.

Last but not least, as mentioned above, ambigol have been shown to inhibit cyclooxygenases. Cyclooxygenases are enzymes belonging to the oxidoreductase enzymatic class. Two related isoforms, COX-1 and COX-2 [336], have been discovered so far and are involved in inflammation processes through the synthesis of prostaglandins from arachidonic acid. Some classical anti-inflammatory molecules are known to target COX. For example, aspirin, which is the most famous COX inhibitor discovered so far, is a nonsteroidal anti-inflammatory drug (NSAID) [337]. For these reasons, ambigol is a promising cyanobacterial anti-inflammatory compound. Nevertheless, further studies are still needed in order to describe its activities and potential unexpected side effects in-depth [338].

#### 5.3.2. Antioxidant Activity

Oxidative stress is widely recognized to be implicated in neurodegenerative diseases [339,340], metabolic disorders [341], hypertension [342], liver diseases [343], and cardiovascular diseases [344]. Thus, antioxidant molecules are required to develop or supplement therapy for reducing the harmful effects of oxidative stress.

According to our review, only four compounds isolated from cyanobacteria show antioxidant properties. As mentioned above, this low number in comparison to cytotoxic or antimicrobial compounds might be due to the fact that this activity has been poorly tested in secondary metabolites and its testing has generally been limited to pigments or molecules implicated in light-harvesting or UV protection. Antioxidant activity has been characterized for the carotenoids, chlorophyll, the mycosporine-like amino acids (MAAs), and the phycobiliproteins such as phycocyanin (Table 10).

Carotenoids are orange pigments that are localized in the thylakoid membrane. They absorb light between 400 and 500 nm and are involved in photosynthesis by transferring energy to chlorophyll through a single-singlet energy transfer mechanism [346,349]. Five carotenoids are found in the majority of cyanobacteria: β-carotene, zeaxanthin, nostoxanthin, echinenone, and canthaxanthin [345]. In addition to their role in light harvesting, carotenoids act as potent photoprotectant molecules and show antioxidant activity through ROS scavenging [345,349] (Table 10).

Chlorophylls are the ubiquitous pigments of photosynthetic organisms. Chlorophyll *a* is the major isoform used by cyanobacteria with most absorbing light at 660 nm [345]. Chlorophylls are mainly involved in photosynthesis, but they have also shown antioxidant activity in vitro via radical scavenging and, on the contrary, singlet oxygen production under high light conditions, which mitigates their potential use as antioxidant therapeutics [345] (Table 10).

Mycosporine-like amino acids (MAAs) are pigments that are widely produced by cyanobacteria (Table 10) and other algae [58,345]. They absorb light in the UV-A and UV-B ranges with a maximum absorbance between 310 and 360 nm [58]. The primary function of MAAs is to protect cells from damage by absorbing UV and to dissipate energy without generating ROS [345,348]. In addition, MAAs show other unique properties. They have been demonstrated to have antioxidant activity through ROS scavenging, are able to protect skin from UV damage, and are involved in osmotic regulation, desiccation, and defense against oxidative and thermal stresses. They are also able to protect fibroblasts against UV-induced cell death [58,348]. Jain et al. (2017) stated that two products containing MAAs have been commercialized as sunscreen agents for cosmetics and for use in plastics, paints, and varnishes as a photo stabilizer [58].

Lastly, as mentioned above, phycocyanins are antioxidant molecules with the ability to scavenge ROS. In addition to their anti-inflammatory activity, this antioxidant property increases the potential of phycocyanins to be used for pharmaceutical applications [325].

### 5.4. Other Metabolites with Potential Beneficial Properties

To close this review on the beneficial activities demonstrated for cyanobacterial metabolites, we highlight a few other compounds that are of potential interest for various fields of application because of their specific features.

For instance, grassystatins-tasiamides constitute a depsipeptide group of related compounds isolated from *Lyngbya* and *Symploca* tropical species [350,351,352,353,354,355,356]. These metabolites have shown protease inhibitory activity against cathepsin D, cathepsin E, and the β-amyloid precursor protein-cleaving enzyme A (BACE1) for tasiamides B and F [350,351] (Table 11). In addition, these compounds have shown moderate or no cytotoxicity at concentrations higher than required for protease inhibitory activity [353,354,356]. Cathepsin D is an aspartic protease that is localized in the lysosome. This enzyme is considered a biomarker of some forms of metastatic breast cancer because of its related overexpression [236]. Cathepsin D has also been shown to promote proliferation and metastasis [236]. Cathepsin E, being also an aspartic protease, is mainly localized in immune system cells and, notably, in antigen-presenting cells [357]. Grassystatin A induces the reduction of antigen presentation in dendritic cells [352], which is correlated with the involvement of cathepsin E in this process and has led to the hypothesis that grassystatin could modulate the immune response. Alzheimer’s disease pathogenesis is mediated by the accumulation of amyloid β peptide (Aβ) in the brain. BACE1 is responsible for Aβ formation by cleaving the amyloid precursor protein (APP). As a result, BACE1 inhibitors could be promising targets for the development of new therapeutics against Alzheimer’s disease [351,358]. Considering these activities, we assume that members of the grassystatins-tasiamides family constitute promising components for the development of antiproliferative agents, immune response modulatory compounds, and therapeutics for Alzheimer’s disease treatment.

During the process of database construction, we noticed that five metabolite families showed a remarkable ability to bind to cannabinoid receptors (CB1 and CB2). These metabolites were grenadamide [359], the semiplenamides [360], serinolamide A [361], mooreamide A [362], and the columbamides [363]. CB1 and CB2 are cell membrane receptors that belong to the endocannabinoid system (ECS), which is an important part of the human physiological system. It is involved in a wide range of different processes, such as brain plasticity, memory, nociception, appetite regulation, the sleep–wake cycle, the regulation of emotions and stress, and addiction. This ubiquity for the regulation of various vital processes makes exogenous CB1 and CB2 ligands attractive as modulators of this system for the management of pain, diabetes, obesity, cancer, epilepsy, or Alzheimer’s disease, or to develop new anxiolytics [364,365]. Columbamides are the most potent CB1/CB2 ligands from cyanobacteria discovered so far (Table 11) [363]. They are linear acyl amides that have been isolated from *Moorea bouillonii* PNG05-198 using a genome mining approach [363]. To date, only the CB1-binding and CB2-binding activity of columbamides has been tested, and other investigations are required in order to look deeper into the activity profiles of these molecules, since they still remain promising compounds for therapeutic developments.

## 6. Conclusions

In this review, all available information concerning the beneficial activities of natural products of cyanobacteria was gathered. To write this review, a molecular database of the various families of metabolites isolated from cyanobacteria was constructed from the systematic analysis of 670 articles. The derived database represents 260 families of metabolites. It groups various types of information concerning the taxonomy of producing strains, the respective chemical classes, the origin strain habitats, and the tested/demonstrated activities for each member of the family, together with the related full references.

According to this review, from the above 300 different genera of cyanobacteria (referenced by the taxonomy published by Komarek et al. in 2014) [27], 90 have, so far, been reported to produce bioactive metabolites. Some of them have been shown to produce a high number of compounds, such as those from the genus *Lyngbya-Moorea*, which includes 85 families of metabolites isolated so far. However, the *Lyngbya* genus is a polyphyletic group and its taxonomy position is under revision. This number might be re-evaluated and distributed within distinctive new genera. The genomes of the producing strains are not available in the majority of cases, whereas Shih et al. (2013) demonstrated the large genomic potential of numerous cyanobacteria thanks to the biosynthetic pathways of metabolites highlighted by genome mining analyses [49]. Therefore, the potential for the discovery of new natural molecules and new biosynthetic pathways from cyanobacteria still remains very important and needs to be systematically explored. 

Cyanobacterial metabolites belong to 10 chemical classes (including peptides, alkaloids, terpenes, and lipids), where most of the families of metabolites are peptide derivatives (above 50% of the families). Fourteen different types of activities can be distinguished for cyanobacterial metabolites (e.g., antimicrobial, lethality, cytotoxicity, and antioxidant). The large majority of the components are cytotoxic (110 families), whereas some activities have only been tested rarely, and their occurrence appears to be weakly demonstrated. Globally, no clear correlation has been observed between chemical classes and the specificity of the respective types of bioactivity. Further studies are needed in order to precisely understand the mechanisms of action of cyanobacterial metabolites, which potentially links bioactivity with structural features in order to support the new hypothesis on the biological function of the production of these components for organisms.

Lastly, 47 metabolites isolated from cyanobacteria that present remarkable interest for diverse fields of application were investigated further in the present literature review. For example, hassallidins, which show specific antifungal activity without antibacterial activity, and scytonemin, which has anti-inflammatory properties with no cytotoxicity, were detailed. These metabolites are potentially useful for the development of new concrete applications for cyanobacterial natural products and illustrate the interest in cyanobacteria as a prolific source of bioactive molecules.

## Figures and Tables

**Table 6:** Cytotoxic metabolites extracted from the database.

Molecule Family	Chemical Classes	Activity	Producing Organisms	References
Tubercidin	Nucleoside	-Cytotoxic -Microtubule stabilizer	*Tolypothrix byssoidea* H-6-2, *Scytonema saleyeriense* var. *indica* CV-14-1, *Plectonema radiosum* DF-6-1, *Tolypothrix distorta* BL-11-2	[198,199,200]
Aurilides	Depsipeptide	-Cytotoxic -Lethal activity -Anti-swarming -Antiprotozoal -Induce loss of microfilament network	*Lyngbya majuscula*, *Okeania* sp., *Lyngbya* sp.	[70,202,203,204,205]
Swinholide-type	Macrolide	-Cytotoxic -Actin microfilament disruption	*Symploca* sp., *Geitlerinema* sp., *Nostoc* sp. UHCC0451, *Phormidium* sp.	[206,207,208]
Anabaenolysins	Lipopeptide	-Cytotoxic -Antifungal -Hemolytic activity -Ability to permeabilize cell membranes	*Anabaena* sp. XPORK 15F, *Anabaena* sp. XSPORK 27C	[209,210]

**Table 7:** Serine protease inhibitor metabolites extracted from the database.

Molecule Family	Chemical Classes	Activity	Producing Organisms	References
Spumigins	Peptide	-Proteases inhibitory activity	*Nodularia spumigena* AV1 & CCY 9414, *Anabaena compacta* NIES-835	[239,240,241]
Cyanopeptolin-like	Depsipeptide	-Protease inhibitory activity -Other enzyme inhibition -Cytotoxic -Lethal -Antibacterial -Antifungal -Antiprotozoal	*Microcystis* sp., *Microcystis aeruginosa*, *Aphanocapsa* sp.; *Microchaete loktahensis*, *Planktothrix agardhii*, *Scytonema hofmanni*, *Lyngbya* sp., *Lyngbya confervoides*, *Lyngbya* spp., *Lyngbya semiplena*, *Microcystis viridis*, *Dichothrix utahensis*, *Nostoc* sp., *Nostoc minutum*, *Planktothrix rubescens*, *Lyngbya majuscula*-*Schizothrix* sp. (Assemblage), *Stigonema* sp., *Symploca* sp., *Symploca hydnoides*, *Nostoc insulare*	[30,31,159,185,186,188,243,244,245,246,247,248,249,250,251,252,253,254,255,256,257,258,259,260,261,262,263,264,265,266,267,268,269,270,271,272,273,274,275,276,277,278,279,280,281,282,283,284,285,286,287,288,289,290,291,292]
Carmaphycins	Peptide	-Protease inhibition -Cytotoxic -Antiprotozoal	*Symploca* sp. WHG NAC15/Dec/08–5	[293,294]

**Table 8:** HDACs inhibitor metabolites extracted from the database.

Molecule Family	Chemical Classes	Activity	Producing Organisms	References
Largazole	Depsipeptide	-Histone deacetylases inhibitor -Cytotoxic -Other enzyme inhibition -Pro-drug	*Symploca* sp.	[311,312,313,314,315,316]
Santacruzamate A	Carboxylic acid derived	-Histone deacetylases inhibitor -Cytotoxic	*Symploca* sp. PAC-19-FEB-10-1	[317]

**Table 9:** Anti-inflammatory metabolites extracted from the database.

Molecule Family	Chemical Classes	Activity	Producing Organisms	References
Aeruginosins	Peptide	-Anti-inflammatory activity -Protease inhibitor -No cytotoxicity	*Microcystis aeruginosa* NIES-98, NIES-298, NIES-101, NIES-89. *Microcystis viridis* NIES-102 *Planktothrix agardhii* CYA 126/8. *Nodularia spumigena* CCY9414. *Nostoc* sp. Lukesova 30/93	[62,321,322,323]
Phycocyanin	Peptide	-Anti-inflammatory -Antioxidant -Specific inhibitor of COX-2 -No lethality	All	[59,64,324,325,326]
Scytonemin	Alkaloid	-Anti-inflammatory -Enzyme inhibition -No cytotoxicity	*Stigonema* sp., *Nostoc punctiforme*, *Anabaena variabilis*, *Anabaena ambigua*, *Aphanocapsa/Synechocystis* sp. (assembly), *Aulosira fertilissima*, *Calothrix* sp., *Calothrix parietina*, *Calothrix crustacea*, *Chlorogloeopsis* sp., *Chroococcidiopsis* sp., *Chroococcus* sp.; *Cylindrospermum* sp., *Diplocolon* sp., *Entophysalis granulos*, *Gloeocapsa* sp., *Hapalosiphon* sp., *Hapalosiphon fontinalis*; *Lyngbya* sp., *Lyngbya aestuarii*, *Nostoc parmelioides*, *Nostoc commune*, *Nostoc microscopium*, *Nostoc pruniforme*, *Phormidium* sp., *Pleurocapsa* sp., *Rivularia* atra, *Rivularia* sp., *Schizothrix* sp., *Scytonema* sp., *Tolyothrix* sp., *Tolypothrix tenni*, *Westiellopsis prolifica*, *Scytonema hoffmani*	[65,327,328,329,330,331]

**Table 10:** Antioxidant metabolites extracted from the database.

Molecule Family	Chemical Classes	Activity	Producing Organisms	References
Carotenoids	Terpenoid	-Antioxidant -Sunscreen	All	[56,345,346]
Chlorophylls	Chlorin	-Photosynthesis -Antioxidant -Pro-oxidant (sensitizer for singlet oxygen production)	All	[57,345]
MAAs	Cyclohexenone linked with an amino acid	-Antioxidant -Sunscreens	*Synechocystis* sp. PCC 6803, *Gloeocapsa* sp. CU-2556, *Aphanothece halophytica*, *Gloeocapsa* sp., *Euhalothece* sp., *Microcystis aeruginosa*, *Arthrospira* sp. CU2556, *Lyngbya* sp. CU2555, *Leptolyngbya* sp., *Phormidium* sp., *Lyngbya* cf. *aestuarii*, *Microcoleus chthonoplastes*, *Microcoleus* sp., *Oscillatoria spongelidae*, *Trichodesmium* spp., *Anabaena* sp., *Anabaena doliolum*, *Anabaena variabilis* PCC 7937, *Nostoc* sp., *Nostoc commune* var. Vaucher, *Nostoc commune*, *Scytonema* sp., *Nostoc punctiforme* ATCC 29133, *Nostoc* sp. HKAR-2 and HKAR-6, *Nodularia baltica*, *Nodularia harveyana*, *Nodularia spumigena*, *Aphanizomenon flos-aquae*, *Chlorogloeopsis* PCC 6912	[58,345,347,348]
Phycocyanin	Peptide	-Anti-inflammatory -Antioxidant -Specific inhibitor of COX-2 -No lethality	All	[59,64,324,325,326]

**Table 11:** Other metabolites extracted from our database with promising biomedical potential.

Molecule Family	Chemical Classes	Activity	Producing Organisms	References
Grassystatins-Tasiamides	Depsipeptide	-Protease inhibitory activity -Cytotoxic -Reduce antigen presentation in dendritic cells	*Lyngbya confervoides*, *Symploca* sp., *Symploca* sp. NHI304, *Lyngbya* sp. NIH399	[350,351,352,353,354,355,356]
Columbamides	Acyl amide	-CB1 and CB2 ligands	*Moorea bouillonii* PNG05-198	[363]

## Supplementary Materials

The following are available online at https://www.mdpi.com/1660-3397/17/6/320/s1. Supplementary Data S1: Amino acid sequences of cyanopeptolin-like family; S2: Cyanobacterial metabolite database; S3: Detailed of the molecules described.
